# Virtual Reality or Augmented Reality as a Tool for Studying Bystander Behaviors in Interpersonal Violence: Scoping Review

**DOI:** 10.2196/25322

**Published:** 2021-02-15

**Authors:** Jia Xue, Ran Hu, Wenzhao Zhang, Yaxi Zhao, Bolun Zhang, Nian Liu, Sam-Chin Li, Judith Logan

**Affiliations:** 1 Factor Inwentash Faculty of Social Work University of Toronto Toronto, ON Canada; 2 Faculty of Information University of Toronto Toronto, ON Canada; 3 University of Toronto Libraries Toronto, ON Canada

**Keywords:** virtual reality, augmented reality, bystander behaviors, interpersonal violence, violent incidents, people’s responses, dating violence, sexual violence

## Abstract

**Background:**

To provide participants with a more real and immersive intervening experience, virtual reality (VR) and/or augmented reality (AR) technologies have been integrated into some bystander intervention training programs and studies measuring bystander behaviors.

**Objective:**

We focused on whether VR or AR can be used as a tool to enhance training bystanders. We reviewed the evidence from empirical studies that used VR and/or AR as a tool for examining bystander behaviors in the domain of interpersonal violence research.

**Methods:**

Two librarians searched for articles in databases, including APA PsycInfo (Ovid), Criminal Justice Abstracts (EBSCO), Medline (Ovid), Applied Social Sciences Index & Abstracts (ProQuest), Sociological Abstracts (ProQuest), and Scopus till April 15, 2020. Studies focusing on bystander behaviors in conflict situations were included. All study types (except reviews) written in English in any discipline were included.

**Results:**

The search resulted in 12,972 articles from six databases, and the articles were imported into Covidence. Eleven studies met the inclusion and exclusion criteria. All 11 articles examined the use of VR as a tool for studying bystander behaviors. Most of the studies were conducted in US young adults. The types of interpersonal violence were school bullying, dating violence, sexual violence/assault, and soccer-associated violence. VR technology was used as an observational measure and bystander intervention program. We evaluated the different uses of VR for bystander behaviors and noted a lack of empirical evidence for AR as a tool. We also discuss the empirical evidence regarding the design, effectiveness, and limitations of implementing VR as a tool in the reviewed studies.

**Conclusions:**

The reviewed results have implications and recommendations for future research in designing and implementing VR/AR technology in the area of interpersonal violence. Future studies in this area may further contribute to the use of VR as an observational measure and explore the potential use of AR to study bystander behaviors.

## Introduction

### Interpersonal Violence and Bystander Intervention

According to the World Health Organization, interpersonal violence refers to “the intentional use of physical force or power, threatened or actual” against individuals who may be family members (including intimate partners), colleagues, acquaintances, or strangers [1]. Drawing on this definition, common forms of interpersonal violence include physical violence, verbal abuse, psychological violence, and sexual assault or harassment [2]. Given the multifaceted and detrimental impact of interpersonal violence on the victims (eg, health and mental health consequences [3-5]), developing effective strategies to prevent violence in the first place becomes crucial. Those who witness violent incidents or potential risks for violence, often known as bystanders, may play an influential role in intervening in the situation, such as providing support to the victims and reporting the incident [6,7].

Empirical studies [8] have focused on examining factors that may influence bystander intervening behaviors, such as bystander self-efficacy [9], skills to intervene [6], and awareness of signs of violence [10]. Using an ecological framework, Banyard [8] suggested taking a multilevel approach, including individual characteristics, community-level influencers, and the context of violence, when considering facilitators and barriers to bystander intervention. Bystander training programs can help potential bystanders intervene appropriately and effectively when witnessing or recognizing signs of violent incidents [11,12]. A wide variety of training methods and their effectiveness have been reported in empirical studies [13-15]. Commonly adopted training methods often include lectures, case scenario discussions, video watching, and other active and experiential learning activities such as the use of theatre [14,16]. These traditional teaching methods have demonstrated their effectiveness, such as in reducing acceptance of violence [14], increasing self-efficacy [17], and increasing the willingness to help [18].

To continue and advance the research on bystander behaviors and the effectiveness of training programs, a growing body of recent studies began to incorporate virtual reality (VR) as a tool for data collection [19-21] or as a central programming component in bystander training interventions [22]. The main advantage of VR lies in its technological advance that affords participants an immersive experiential environment. More specifically, VR surrounds individuals with a 360-degree computer-generated immersive environment, substituting real-life sensory input (predominantly visual, auditory, and tactile) using wall projections (eg, Cave Automatic Virtual Environment [CAVE]) or head-mounted display devices (eg, Oculus Rift) [23]. Besides, in a VR environment, there are one or more trackers in the room and/or attached to the user’s body parts to track the user’s head and body positions and movements [24]. Similar to what VR may offer, augmented reality (AR) allows creating an environment that integrates both a simulated virtual scenario and a real-world physical setting. AR superimposes digital materials over what individuals perceive in the real world [25]. In an AR environment, people can perceive and interact with virtual and physical objects, allowing for an extended real-life experience. The most common AR delivery platforms are mobile devices (eg, in Pokémon GO, one can see and play with virtual comic characters in their physical environment through the phone camera), and technology companies are experimenting with AR glasses, such as Microsoft HoloLens and Apple AR glasses. In short, VR and AR are on the reality-virtuality continuum of Milgram and Kishino [26], ranging from physical reality on one end to a completely virtual environment (ie, VR) on the other, with a blend of physical and virtual environments (ie, AR) in between [27].

The integration of VR or AR may provide new methodological and training strategies to bystander behavior research. For instance, VR provides an immersive experience that creates presence [28], shortens bystanders’ psychological distance to the conflict scenarios [22], and invites bystanders to behave as if the environment is real [29]. As such, VR unites the generalizability of standardized scenarios and ecological validity by allowing bystanders to behave naturally in a controlled immersive environment [20,30]. Further, as a VR environment allows for behavioral measurements (ie, tracking), researchers can examine verbal and physical bystander behaviors rather than solely relying on self-reports, which furthers the ecological validity [20,31]. While VR simulates real-life interpersonal conflicts in a virtual environment, AR superimposes additional information on the real environment where the conflicts take place. AR has shown effectiveness in training interpersonal behaviors by digitally providing extra information about social cues [32]. In addition, mobile AR requires only a smartphone to operate and is an affordable bystander training platform for many organizations and individuals.

### Aim of the Study

This review aims to comprehensively assess current evidence from empirical studies in social science and computer science databases and evaluate the use of VR or AR as a tool for studying bystander behaviors in the domain of interpersonal violence research. The reviewed results have implications and recommendations for future research in designing and implementing VR/AR technology in the area of interpersonal violence. We will examine the following questions: (1) What are the characteristics of studies using VR and/or AR in examining bystander behaviors? (2) What types of violent incidents were used in a VR and/or AR-simulated environment? (3) How are VR and/or AR used in assessing bystander behaviors or interventions? (4) What are the implications and limitations of implementing VR and/or AR as a tool in the articles?

## Methods

### Design

We conducted a scoping review to address the aforementioned research questions. Arksey and O’Malley’s [33] framework guided the study, including “identifying the research questions; identifying relevant studies; study selection; charting the data; and collating, summarizing, and reporting the results.” This review used the PRISMA-ScR (Preferred Reporting Items for Systematic Reviews and Meta-Analyses Extension for Scoping Reviews) checklist [34]. The team consisted of members in social work, information science, data science, and user experience design.

### Identifying Relevant Studies

Two team members who were librarians (JL and SCL) developed a primary search strategy in APA PsycInfo (Ovid) using a combination of text words and controlled vocabulary. The structure of the search was such that results could mention either VR or AR as long as it included bystanders (Multimedia Appendix 1). Given the long list of search terms, we only listed several examples, such as “avatars,” “virtual reality,” “helmet-mounted display,” “Oculus Quest,” “augmented reality,” “bystander,” “helping behaviors,” and “witness.” This primary search strategy was validated against an a priori test set of articles. It was peer-reviewed by another librarian, not on the team, using the Peer Review of Electronic Search Strategies (PRESS) framework. The search was then translated into the following five other databases, which were searched separately: Criminal Justice Abstracts (EBSCO), Medline (Ovid), Applied Social Sciences Index & Abstracts (ProQuest), Sociological Abstracts (ProQuest), and Scopus. All of the search queries are available in Multimedia Appendix 1. A language limit of English was applied in databases where the search retrieved over 100 results. Search results were downloaded on April 15, 2020, and uploaded to Covidence for deduplication and screening.

### Study Selection

#### Inclusion Criteria

The team established the inclusion criteria before the screening. The team participated in training such as examining the sample search results and discussing the inclusion criteria as follows: (1) the article focused on bystanders in a violence situation; (2) the article used digital virtual simulations, such as VR and AR; and (3) the article was written in English.

#### Exclusion Criteria

Articles were excluded from the review if they (1) only examined bystanders in nonconflict-related situations (eg, sudden heart attack); (2) only considered real settings, nondigital virtual settings, or non-VR/AR–related digital settings (any study only using nonimmersive virtual environments [eg, experienced through digital screens] was excluded); (3) were published in other languages; and (4) were any type of a review study, synthesis, book chapter, editorial, letter to the Editor, commentary, or opinion piece.

### Selection Procedure and Search Results

**Figure 1:**
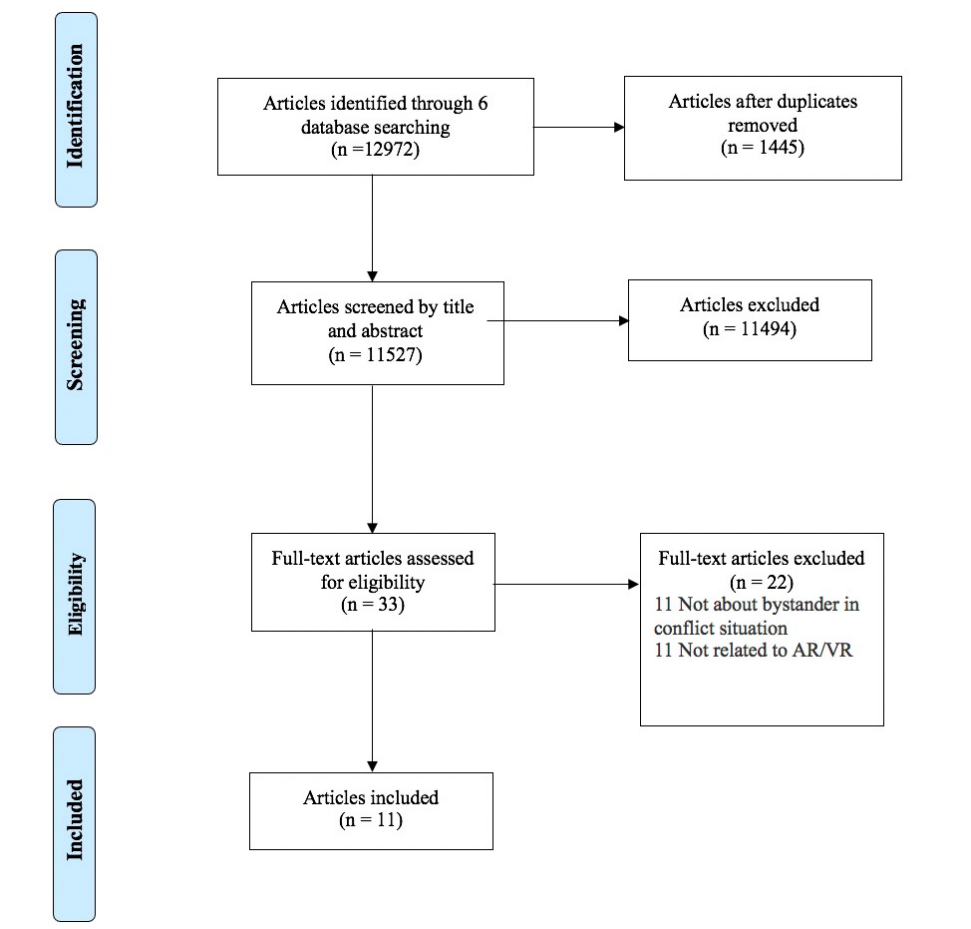
Selection procedure and search results. AR: augmented reality; VR: virtual reality.

As shown in Figure 1, the librarians imported 12,972 articles from six databases into Covidence. After removing 1445 duplicates, we had 11,527 articles for the title and abstract screening. First, guided by the team chair, five research assistants participated in title and abstract screening training using a random sample of 100 articles from the database. Research assistants discussed the disagreements during the training. The training was repeated twice, and we reached an interrater reliability of 100%. Second, each article was screened randomly by two out of the five research assistants. Each research assistant was assigned to review the title and abstract of 4586 articles. Third, conflict results were discussed and resolved by the team chair. We removed 11,494 ineligible articles, and a total of 33 articles remained for the full-text assessment. Fourth, each article was reviewed randomly by two out of the five research assistants. When two research assistants had any conflict, the whole team discussed the conflicts and reached a consensus. Finally, 22 articles failed to meet the inclusion criteria of the full-text screening (eg, unrelated to bystanders, unrelated to violence situations, and unrelated to VR/AR). Fifth, the team also conducted a round of hand search for key journals (eg, based on the references of the selected articles). The hand search did not add any new articles to the final data set. Therefore, our final sample for the review included 11 studies.

### Charting the Data

The stage of charting the data was also called “data extraction” in systematic reviews. We developed an information form to extract contents from the included articles (Tables S1, S2, and S3 in Multimedia Appendix 2), such as study location, study design, aims of the study, methodology, outcome measures, and important results. To ensure a reliable extraction process, each included article was reviewed by two authors independently, and a third author resolved any disagreements. The intercoder reliability score was excellent (Cohen κ >90% for all groups). The review process took 3 months and completed in June 2020.

### Collating, Summarizing, and Reporting the Results

According to Arksey and O’Malley’s [33] framework, unlike systematic reviews, our scoping review did not synthesize and evaluate the evidence quality in the studies. Instead, the scoping review summarized the research methods, sample sizes, participants, measures, geographical locations, and outcomes of the studies to report the main areas of interest and research gaps.

## Results

### Review Results and Summary of the Main Characteristics of the Studies

Five research assistants assessed each of the selected articles’ contents and quality, and extracted and summarized the data in response to our research aim. We have charted and presented the results in Tables S1, S2, and S3 in Multimedia Appendix 2. Table S1 in Multimedia Appendix 2 presents the characteristics of the studies, including country, type of violence, participants, age, and study design. Table S2 in Multimedia Appendix 2 shows the study findings, including research questions, measurements, summary of key findings, strengths, and limitations. Table S3 in Multimedia Appendix 2 presents the design, equipment of the VR in the articles, length of the VR/AR experience, and descriptions of the scenarios.

Of the 11 studies included in the review, seven were conducted in the United States, two were conducted in the United Kingdom, one was conducted in Spain, and one did not specify the country where the research was carried out. All studies used VR as a tool to simulate certain situations of violence, and we did not find any studies that employed AR techniques. Nine studies used VR to assist observational research, and the remaining two applied VR in bystander intervention programs [22,35]. While most studies were operationalized as an experiment with participants incorporating a simulated VR environment, three studies were randomized controlled trials [20,30,35]. Different violent incident scenarios were used across these studies, including school bullying behaviors (including relational, verbal, and/or physical violence) among student peers [22,35]; sexual assault incidents that took place in dating or a potential dating relationship [20,21,35,36]; a combination of different types of assaults among friends or acquaintances [30]; and verbal and physical assaults that occurred among strangers [19,29,31,37]. In addition to the victim-aggressor relationship, we examined the relationship between victims and bystanders simulated in the VR scenarios, including school peers (n*=*2) [22,35], friends (n*=*5) [20,21,30,36,38], and strangers (n*=*4) [19,29,31,37]. Regarding study participants, four studies recruited middle or high school students [20,22,35,38], three studies sampled undergraduate students [21,30,36], and the four remaining studies included participants who identified as soccer fans supporting different teams [19,29,31,37].

#### Bullying Behaviors Among School Peers

Two studies focused on bullying behaviors among student peers in middle or high schools. They were the only two studies that included VR as a central component to deliver bullying prevention training [22,35]. Specifically, through a pseudorandomized trial, Ingram et al [22] examined whether a bullying prevention curriculum enhanced by the use of VR reduces students’ bullying behaviors, including traditional bullying, cyberbullying, and relational aggression and whether the VR-enhanced intervention increases bystanders’ willingness to intervene and school belonging. The study recruited 118 students from two middle schools in the United States (46 in VR-enhanced training and 72 in non-VR/regular training). To provide an immersive user experience, a set of customized VR scenarios was simulated using Google goggles [39,40]. Although the VR-enhanced condition did not reduce bullying behaviors, individuals in the VR group reported greater empathy from pretest to posttest than the non-VR group. Specifically, mediated by empathy, students in the VR condition showed decreased bullying perpetration and increased wiliness to intervene as bystanders than the control group. This study’s findings suggest the promise of applying VR in school bullying prevention programs to enhance effectiveness. However, the study by McEvoy et al [35] provided somewhat different and more nuanced findings of the effectiveness of VR.

McEvoy et al [35] also conducted a randomized experimental study that aimed to compare the following three conditions: (1) a customized VR condition, (2) a noncustomized VR condition, and (3) a video condition, in order to examine whether including VR in a bullying prevention program is more effective than a less immersive video-based intervention. Seventy-eight college students participated in the study and randomly joined one of the following three conditions: (1) a 30-second video featuring a female student being verbally and physically bullied by two other female students in the school, (2) a customized VR experience based on the video in which the victim had the participants’ university logo printed on her shirt, and (3) a noncustomized VR experience based on the video in which the victim had no school logo on the shirt. This study used a video from the “Be More than a Bystander campaign” by the Ad Council and the US Department of Health and Human Services [35]. The authors used Unity, a VR development software, and delivered the VR video by Oculus Rift. The results showed that participants in the video group reported higher empathy levels than those in the other two groups, and there were no differences in other measures across the three conditions. The data analysis from a follow-up focus group showed that the VR simulations needed additional interactive features to be effective. To enhance the effectiveness of incorporating VR techniques in bullying intervention programs, program developers should focus on enhancing the realism of VR-simulated environments and their autonomy in their immersive training experience [35].

#### Sexual Assault in Dating Relationships

Of the five studies that included sexual assault (or risks of sexual assault) in their VR-enhanced environments [20,21,35,36,38], two created sexual assault scenarios that also incorporated other types of violence [30,36]. For instance, in the study by Sargent et al [30] involving sexual assault, physical violence, stalking, and coercive controlling behavior were also simulated as part of the VR experience. In all five studies, VR was designed to assist the research rather than to develop a bystander intervention program. For instance, Sargent et al [30] designed VR simulations to determine whether VR can be used as a valid tool for assessing adolescent resistance to antisocial peer pressure. The study findings did show the psychometrical soundness of the VR simulations in research. Other studies also utilized the experiential and immersive nature of VR to collect behavioral data from participants, complementing the traditional self-report surveys. For instance, Jouriles et al [20] conducted a randomized controlled trial with 165 high school students (85 in the intervention) to assess whether the video bystander program TakeCARE increases bystander behaviors. Instead of solely relying on self-report measures to assess bystander intervening behaviors, Jouriles et al [20] also used VR simulations to collect observational data of bystander behaviors to evaluate the durability effects of TakeCARE at postintervention and 6-month follow-up assessments. Wearing goggles, all participants in the study took part in four out of nine immersive VR simulations, all of which provided them opportunities to intervene in sexual assault in a dating or potential dating relationship. Results confirmed the effectiveness of the TakeCARE bystander program to increase bystander behaviors.

Similarly, to examine whether having adverse consequences of being an active bystander was related to lower efficacy for intervening and less effective bystander behavior, Krauss et al [36] simulated a VR environment in which student participants were given opportunities to intervene in sexual and relationship violence on campus. A total of 299 first-year undergraduate students participated in a laboratory-based assessment. Among them, 65% of the students received 20-minute bystander training (TakeCARE) given by the university before the study. In the assessment, the students participated in three 2 to 3-minute VR simulations in which they had opportunities to help the victims. Students used Oculus Rift goggles in the simulations. The results showed that negative consequences of previous bystander behaviors (being physically hurt/getting into trouble resulting from helping someone at risk of sexual assault) predicted lower bystander efficacy and effectiveness. The study also found that bystander training decreased the negative consequences of the participants. Although a limited number of different types of adverse effects were included in the analysis, the study findings indicated the importance of addressing the potential negative consequences bystanders may face or fear and educating students on how to perform safe interventions.

#### Verbal and Physical Assault Among Strangers

Verbal conflicts and physical assault were the focus of four studies [19,29,31,37]. All the simulated violence scenarios occurred among strangers in a public setting, such as in a bar. All these studies used VR to create an observational research environment to collect participants’ behavioral data in simulated violent scenarios as bystanders. For example, Hortensius et al [29] examined whether reflexive and reflective behavioral responses to an emergency are related to later helping behavior in a violent conflict. Twenty-nine male FC Barcelona supporters (age range 18-29 years) participated in the study. They were placed in a VR-enhanced simulated conversation with the virtual victim for about 2 minutes and then witnessed a series of conflicts for 135 seconds. The technologies used in the study included the XVR programming platform [41], the virtual characters animated with HALCA software [42], and a CAVE system [43]. The results showed that the increased helping behavior was associated with a faster response to an emergency in a low cognitive load condition.

## Discussion

### Overview

To the best of our knowledge, our study is the first comprehensive review that evaluates studies using VR or AR as a tool for studying bystander behaviors in the domain of interpersonal violence research. This review screened 12,972 articles and assessed 11 qualified articles in full text. Evaluations of 11 eligible studies provided insights for VR/AR technology and their applications in the domain of interpersonal violence research, including VR as an observational measure, VR as an intervention tool for bystander programs, AR as a tool for the study of bystander behaviors, and the equipment and implementation of a VR system.

### VR as an Observational Measure

For decades, the methodological approach for studying bystander behaviors has been participant self-reported questionnaires in response to written descriptions or videos. Our review found that studies used VR technology as a tool for observational measures (Table S1 in Multimedia Appendix 2). VR technology is a tool to present simulated scenarios. Participants are placed in immersive simulated scenarios in which their bystander behaviors can be studied. For example, participants are given opportunities to intervene in imminent violence/assault as an active bystander in these simulations (eg, girls drunk at a party, physical aggression between dating partners, and unwanted sexual activity at a party). Their bystander behaviors in the VR simulations are audio recorded, observed, and rated by coders on a 7-point scale, indicating their reactions and levels of attempting to intervene in the possible sexual or dating violence. VR provides participants an immersive virtual environment in which they genuinely interact with the avatars, which reduces the Hawthorne effect [44] (participants do not reveal their real behaviors when they realize they are being observed and studied [45]). Current evidence shows that immersive VR offers an under control environment created by computers (eg, perpetrators are made smaller in size and weaker) and, at the same time, ensures that people respond realistically. Thus, the design and implementation of these simulated environments are essential to spark any emotional, cognitive, or behavioral responses from the participants [19]. VR has opened windows of opportunities for innovative multimethods in the study of bystander behaviors in interpersonal violence situations. Further studies should continue to explore how to further improve the reliability and validity of VR as an emerging tool for observational measures.

### VR as an Intervention Tool for Bystander Programs

Two studies examined whether the use of VR enhances existing bystander bullying programs [22,35]. Immersive VR provides an experimental environment that mirrors real-life situations [19]. VR-enhanced bullying scenarios are designed for participants (adolescents) to simulate real-life bullying situations. For example, the participant wears an Oculus Rift RV headset and headphones and experiences the designed scenarios (witnessing a female student being verbally and physically bullied by other students in the school hallway between classes) [35] (Table S3 in Multimedia Appendix 2). The contents of scenarios are designed to resemble bullying prevention program videos (eg, audio and message screen) or be informed by the empirical literature [22]. The benefits of VR over video curriculum as a tool for violence prevention are suggested. Unlike the video curriculum, participants can look around in immersive VR simulations, even though they cannot interact with the avatars. In addition to the video curriculum, the VR-enhanced prevention tool allows participants to experience different perspectives in the simulation, such as being bystanders, being victims, and being adults to intervene. VR is an ecologically valid environment in which researchers can overcome ethical issues in violence studies and prevent potential real physical danger to participants [19].

### AR as a Tool for Bystander Behaviors

Our review did not find any empirical studies that assessed the use of AR for bystander behaviors in interpersonal violence situations. AR enables integrating physical and virtual elements into one view by allowing participants to merge the virtual component into the real physical world. AR is different from VR, and it enables participants to be in an immersive virtual world [46]. Previous research in AR indicates that challenges exist in the social acceptance of AR, such as privacy concerns [47,48]. For example, the subtle design of the Google glass makes bystanders around feel privacy destruction and intrusion [49,50]. This review suggests that future studies may examine the feasibility and effectiveness of using AR technology to study bystander behaviors in the domain of interpersonal violence research.

### Equipment and Implementation of the VR System

The results of the reviewed articles suggest that future studies should give attention to what technology is being used as an experimental tool and an observational measure, and how VR platforms are set up because these could affect the results. We have identified several key features of the design and equipment for using VR to study bystander behaviors in interpersonal violence research. These include designed virtual avatars and VR scenarios, a CAVE-like system (automatic virtual environment), glasses for immersive 3D vision, including headphones and a microphone, a head tracker, a video camera for recording, and a programming platform (or simulations created and coded by the authors).

In our review, only four studies [19,29,31,37] used a CAVE-like system. For example, Rovira et al [19] used Trimension ReacTor, which has three back-projected acrylic screens (front, left, and right walls; 3 m × 2.2 m) and a floor screen projected from a ceiling-mounted projector (3 m × 3 m) [51,52]. The CAVE system used in these studies has variations in pixel resolution and Hertz (monitor’s refresh rate, 60 Hz means the monitor refreshes its image 60 times per second), which have impacts on participants’ intervention behaviors [37].

Without employing a sophisticated CAVE-like system, head-mounted display devices can be used only to design a VR-based project for studying and measuring bystander behaviors. Different glasses are used to synchronize with the projectors in the VR system, such as Oculus Rift [30,35,36], Crystal Eyes shutter glasses [19,29,31,37], and Daydream goggles [22]. Three studies did not indicate the brand of the goggles used [20,21,38].

### Limitations

There are several limitations in this review. First, we only included articles using VR and/or AR as a tool for studying bystander behaviors and therefore excluded studies that examined the engagement of violence on these platforms, such as harassment in social VR [53] and violence in video games [54]. Second, there were variations in the design and equipment of VR in these articles, making it challenging to perform cross-study comparisons. Despite these limitations, our study is the first to review the use of VR as a tool for bystander behaviors in interpersonal violence research.

### Future Research: Recommendations for the Use of VR in Interpersonal Violence Research

Further studies are needed to provide a rich understanding of the use of VR in the domain of interpersonal violence research. First, future studies should further compare the effectiveness of VR as an intervention tool with the well-established bystander intervention curriculum based on videos. Second, the cost of VR may prevent its widespread implementation. Future studies should further explore potential low-cost VR designs and equipment. Third, future research may consider collaborating with human-computer interaction experts to examine whether technology’s quality is associated with efficacy. For example, although current evidence cannot quantify the effects of display resolution and luminance on people’s responses, detailed facial expressions of the avatars (eg, 1400 × 1050 pixels, 100-Hz refresh rate, 3150 lumens, and digital light processing projectors) encourage more empathic bystander responses [37]. Such recommendations can help determine whether the development and implementation of VR technology are cost-effective in research to increase bystanders’ intent to intervene in violent situations. Fourth, future research should explore the variables that mediate the use of VR, such as psychological distance in bullying [22], photorealistic graphics [35], participants’ familiarity with VR technology, and variations across administrations [21]. Fifth, given the small sample size in all reviewed articles, no evidence was provided with implementation in adults and older adults. Future studies may consider expanding the age range to a broader population to increase the generalizability of the findings.

### Conclusion

There remain considerable gaps in the literature regarding the use of VR technology, notably AR, as a tool for studying bystander behaviors in the domain of interpersonal violence research. The current evidence suggests the effectiveness of VR as an observational measure in addition to self-reported questionnaires and as an intervention tool compared with video-only bystander programs. A limited number of studies exist, and it justifies further research efforts in this area.

## Appendix

Multimedia Appendix 1 Complete search strategy.

Multimedia Appendix 2 Summaries of the studies, research findings, and virtual reality/augmented reality designs.

## Abbreviations

AR: augmented reality
CAVE: Cave Automatic Virtual Environment
VR: virtual reality
