# Supplementary material for: Virtual Reality or Augmented Reality as a Tool for Studying Bystander Behaviors in Interpersonal Violence: Scoping Review
Source: J Med Internet Res. 2021 Feb 15;23(2):e25322. doi: 10.2196/25322 (PMC7920754; doi:10.2196/25322)
Supplement: Multimedia Appendix 1 [file jmir_v23i2e25322_app1.docx]

# **Appendix: Complete search strategies**

##

## **APA PsycInfo (Ovid)**

**This query retrieved 714 results on April 15, 2020.**

1. avatars/ or virtual reality/ or virtual reality exposure therapy/

2. ((virtual or digital or mixed) adj2 (realit* or environment* or simulation*)).tw.

3. (human adj2 interface*).tw.

4. (VR or avatar* or "data glove*" or "haptic interface*" or "helmet mounted display*" or HMD or telepresence or "Google Cardboard" or "Google Daydream" or "Google VR" or "HTC Vive" or "Oculus Go" or "Oculus Mobile" or "Oculus Quest" or "Oculus Rift" or "Oculus SDK" or "Oculus Windows SDK" or "OpenV SDK" or OSVR or "Playstation VR" or "Samsung Gear VR" or "SteamVR SDK" or VRTK).tw.

5. or/1-4 [virtual reality]

6. augmented reality/

7. ((augmented or extended or enhanced or altered) adj2 (realit* or environment* or simulation*)).tw.

8. (AR or ARCore or ARKit or HoloLens or Maxst or Vuforia or Wikitude or Windows mixed reality).tw.

9. or/6-8 [augmented reality]

10. bystander effect/ or observers/ or witnesses/

11. (beholder* or bystander* or "by-stander*" or cyberstander* or "helping behavi*" or observer* or onlooker* or passerby or passersby or passiv* or spectator* or "third part*" or "3rd part*" or watcher* or witness* or eyewitness*).tw.

12. or/10-11 [bystanders]

13. 5 or 9

14. 12 and 13

15. limit 14 to (chinese or english)

## **Criminal Justice Abstracts (EBSCO)**

**This query retrieved 42 results on April 15, 2020.**

( TI ( ((virtual OR digital OR mixed) N2 (realit* OR environment* OR simulation*)) OR (human N2 (interface*)) OR VR OR avatar* OR "data glove*" OR "haptic interface*" OR "helmet mounted display*" OR HMD OR telepresence OR "Google Cardboard" OR "Google Daydream" OR "Google VR" OR "HTC Vive" OR "Oculus Go" OR "Oculus Mobile" OR "Oculus Quest" OR "Oculus Rift" OR "Oculus SDK" OR "Oculus Windows SDK" OR "OpenV SDK" OR OSVR OR "Playstation VR" OR "Samsung Gear VR" OR "SteamVR SDK" OR VRTK ) OR AB ( ((virtual OR digital OR mixed) N2 (realit* OR environment* OR simulation*)) OR (humanN2 (interface*)) OR VR OR avatar* OR "data glove*" OR "haptic interface*" OR "helmet mounted display*" OR HMD OR telepresence OR "Google Cardboard" OR "Google Daydream" OR "Google VR" OR "HTC Vive" OR "Oculus Go" OR "Oculus Mobile" OR "Oculus Quest" OR "Oculus Rift" OR "Oculus SDK" OR "Oculus Windows SDK" OR "OpenV SDK" OR OSVR OR "Playstation VR" OR "Samsung Gear VR" OR "SteamVR SDK" OR VRTK ) OR SU ("mixed reality" OR "three-dimensional imaging" OR "virtual reality" )

OR

TI ( (((augmented OR extended OR enhanced OR altered) N2 (realit* OR environment* OR simulation*)) OR "AR" OR ARCore OR ARKit OR HoloLens OR Maxst OR Vuforia OR Wikitude OR "Windows mixed reality") ) OR AB ( (((augmented OR extended OR enhanced OR altered) N2 (realit* OR environment* OR simulation*)) OR "AR" OR ARCore OR ARKit OR HoloLens OR Maxst OR Vuforia OR Wikitude OR "Windows mixed reality") ) OR SU "augmented reality" )

AND

( TI ( (beholder* OR bystander* OR "by-stander*" OR cyberstander* OR "helping behavi*" OR observer* OR onlooker* OR passerby OR passersby OR passiv* OR spectator* OR "third part*" OR "3rd part*" OR watcher* OR witness* OR eyewitness*) ) OR AB ( (beholder* OR bystander* OR "by-stander*" OR cyberstander* OR "helping behavi*" OR observer* OR onlooker* OR passerby OR passersby OR passiv* OR spectator* OR "third part*" OR "3rd part*" OR watcher* OR witness* OR eyewitness*) ) OR SU ( "bystander effect (psychology)" OR "bystander involvement" OR passersby OR "passivity (psychology)" OR spectators" OR "third parties (law)" OR "third party liability" OR witnesses ) )

## **Medline (Ovid)**

**This query retrieved 3,255 results on April 15, 2020.**

1. ((virtual or digital or mixed) adj2 (realit* or environment* or simulation*)).tw,kf.

2. (human adj2 interface*).tw,kf.

3. (VR or avatar* or "data glove*" or "haptic interface*" or "helmet mounted display*" or HMD or telepresence or "Google Cardboard" or "Google Daydream" or "Google VR" or "HTC Vive" or "Oculus Go" or "Oculus Mobile" or "Oculus Quest" or "Oculus Rift" or "Oculus SDK" or "Oculus Windows SDK" or "OpenV SDK" or OSVR or "Playstation VR" or "Samsung Gear VR" or "SteamVR SDK" or VRTK).tw,kf.

4. virtual reality/ or "imaging, three-dimensional"/ or "man-machine systems"/

5. or/1-4 [virtual reality]

6. ((augmented or extended or enhanced or altered) adj2 (realit* or environment* or simulation*)).tw,kf.

7. (AR or ARCore or ARKit or HoloLens or Maxst or Vuforia or Wikitude or Windows mixed reality).tw,kf.

8. augmented reality/

9. or/6-8 [augmented reality]

10. (beholder* or bystander* or "by-stander*" or cyberstander* or "helping behavi*" or observer* or onlooker* or passerby or passersby or passiv* or spectator* or "third part*" or "3rd part*" or watcher* or witness* or eyewitness*).tw,kf.

11. helping behavior/

12. or/10-11 [bystanders]

13. 5 or 9

14. 12 and 13

15. limit 14 to (chinese or english)

## **Applied Social Sciences Index & Abstracts (ProQuest)**

**This query retrieved 60 results on April 15, 2020.**

[STRICT] (TIAB(((virtual OR digital OR mixed) N/2 (realit* OR environment* OR simulation*)) OR (human N/2 interface*) OR VR OR avatar* OR "data glove*" OR "haptic interface*" OR "helmet mounted display*" OR HMD OR telepresence OR "Google Cardboard" OR "Google Daydream" OR "Google VR" OR "HTC Vive" OR "Oculus Go" OR "Oculus Mobile" OR "Oculus Quest" OR "Oculus Rift" OR "Oculus SDK" OR "Oculus Windows SDK" OR "OpenV SDK" OR OSVR OR "Playstation VR" OR "Samsung Gear VR" OR "SteamVR SDK" OR VRTK) OR

IF(((virtual OR digital OR mixed) N/2 (realit* OR environment* OR simulation*)) OR (human N/2 (interface*)) OR VR OR avatar* OR "data glove*" OR "haptic interface*" OR "helmet mounted display*" OR HMD OR telepresence OR "Google Cardboard" OR "Google Daydream" OR "Google VR" OR "HTC Vive" OR "Oculus Go" OR "Oculus Mobile" OR "Oculus Quest" OR "Oculus Rift" OR "Oculus SDK" OR "Oculus Windows SDK" OR "OpenV SDK" OR OSVR OR "Playstation VR" OR "Samsung Gear VR" OR "SteamVR SDK" OR VRTK) OR

MAINSUBJECT("human-computer interface" OR "man-machine interfaces" OR "three dimensional forms" OR "virtual reality") OR

TIAB(((augmented OR extended OR enhanced OR altered) NEAR/2 (realit* OR environment* OR simulation*)) OR AR OR ARCore OR ARKit OR HoloLens OR Maxst OR Vuforia OR Wikitude OR "Windows mixed reality") OR

IF(((augmented OR extended OR enhanced OR altered) NEAR/2 (realit* OR environment* OR simulation*)) OR AR OR ARCore OR ARKit OR HoloLens OR Maxst OR Vuforia OR Wikitude OR "Windows mixed reality")

)

AND

(

TIAB(beholder* OR bystander* OR "by-stander*" OR cyberstander* OR "helping behavi*" OR observer* OR onlooker* OR passerby OR passersby OR passiv* OR spectator* OR "third part*" OR "3rd part*" OR watcher* OR witness* OR eyewitness*) OR

IF(beholder* OR bystander* OR "by-stander*" OR cyberstander* OR "helping behavi*" OR observer* OR onlooker* OR passerby OR passersby OR passiv* OR spectator* OR "third part*" OR "3rd part*" OR watcher* OR witness* OR eyewitness*) OR

MAINSUBJECT(bystanders OR eyewitnesses OR "helping behavior" OR observers OR passivity OR spectators OR "third parties" OR witnesses)

)

## **Sociological Abstracts (ProQuest)**

**This query retrieved 92 results on June 25, 2020.**

(TIAB(((virtual OR digital OR mixed) N/2 (realit* OR environment* OR simulation*)) OR (human N/2 interface*) OR VR OR avatar* OR "data glove*" OR "haptic interface*" OR "helmet mounted display*" OR HMD OR telepresence OR "Google Cardboard" OR "Google Daydream" OR "Google VR" OR "HTC Vive" OR "Oculus Go" OR "Oculus Mobile" OR "Oculus Quest" OR "Oculus Rift" OR "Oculus SDK" OR "Oculus Windows SDK" OR "OpenV SDK" OR OSVR OR "Playstation VR" OR "Samsung Gear VR" OR "SteamVR SDK" OR VRTK) OR

IF(((virtual OR digital OR mixed) N/2 (realit* OR environment* OR simulation*)) OR (human N/2 interface*) OR VR OR avatar* OR "data glove*" OR "haptic interface*" OR "helmet mounted display*" OR HMD OR telepresence OR "Google Cardboard" OR "Google Daydream" OR "Google VR" OR "HTC Vive" OR "Oculus Go" OR "Oculus Mobile" OR "Oculus Quest" OR "Oculus Rift" OR "Oculus SDK" OR "Oculus Windows SDK" OR "OpenV SDK" OR OSVR OR "Playstation VR" OR "Samsung Gear VR" OR "SteamVR SDK" OR VRTK) OR

MAINSUBJECT("Virtual reality") OR

(TIAB(((augmented OR extended OR enhanced OR altered) NEAR/2 (realit* OR environment* OR simulation*)) OR AR OR ARCore OR ARKit OR HoloLens OR Maxst OR Vuforia OR Wikitude OR "Windows mixed reality") OR

IF(((augmented OR extended OR enhanced OR altered) NEAR/2 (realit* OR environment* OR simulation*)) OR AR OR ARCore OR ARKit OR HoloLens OR Maxst OR Vuforia OR Wikitude OR "Windows mixed reality"))

)

AND

(

TIAB(beholder* OR bystander* OR "by-stander*" OR cyberstander* OR "helping behavi*" OR observer* OR onlooker* OR passerby OR passersby OR passiv* OR spectator* OR "third part*" OR "3rd part*" OR watcher* OR witness* OR eyewitness*) OR

IF(beholder* OR bystander* OR "by-stander*" OR cyberstander* OR "helping behavi*" OR observer* OR onlooker* OR passerby OR passersby OR passiv* OR spectator* OR "third part*" OR "3rd part*" OR watcher* OR witness* OR eyewitness*) OR

MAINSUBJECT(“Helping behavior” OR Observation OR Passiveness OR Spectators OR Witnesses)

)

## **Scopus**

**This query retrieved 8,794 results on April 15, 2020.**

TITLE-ABS-KEY ( ( ( ( virtual OR digital OR mixed ) W/2 ( realit* OR environment* OR simulation* ) ) OR ( human W/2 interface* ) OR vr OR avatar* OR "data glove*" OR "haptic interface*" OR "helmet mounted display*" OR hmd OR telepresence OR "Google Cardboard" OR "Google Daydream" OR "Google VR" OR "HTC Vive" OR "Oculus Go" OR "Oculus Mobile" OR "Oculus Quest" OR "Oculus Rift" OR "Oculus SDK" OR "Oculus Windows SDK" OR "OpenV SDK" OR osvr OR "Playstation VR" OR "Samsung Gear VR" OR "SteamVR SDK" OR vrtk ) OR ( ( ( augmented OR extended OR enhanced OR altered ) W/2 ( realit* OR environment* OR simulation* ) ) OR ar OR arcore OR arkit OR hololens OR maxst OR vuforia OR wikitude OR "Windows mixed reality" ) ) AND TITLE-ABS-KEY ( beholder* OR bystander* OR "by-stander*" OR cyberstander* OR "helping behavi*" OR observer* OR onlooker* OR passerby OR passersby OR passiv* OR spectator* OR "third part*" OR "3rd part*" OR watcher* OR witness* OR eyewitness* ) AND ( LIMIT-TO ( LANGUAGE , "English" ) OR LIMIT-TO ( LANGUAGE , "Chinese" ) )
