# Supplementary material for: Virtual Reality or Augmented Reality as a Tool for Studying Bystander Behaviors in Interpersonal Violence: Scoping Review
Source: J Med Internet Res. 2021 Feb 15;23(2):e25322. doi: 10.2196/25322 (PMC7920754; doi:10.2196/25322)
Supplement: Multimedia Appendix 2 [file jmir_v23i2e25322_app2.docx]

**Table S1.** Summary of the studies.

| Study | Country | Victim-aggressor relationship | Victim-bystander relationship | Type of violence | Participants | Age | Study design | The use of VR as a tool |
| --- | --- | --- | --- | --- | --- | --- | --- | --- |
| [22] Ingram et al., (2019) | USA | School peers | School peers | Relational aggression in traditional and cyber forms | 118 7^th^ and 8^th^-grade students from two Midwest middle schools in the US | Range: 11 – 14  Mean =12.5, SD=0.61 | Pseudo-randomized pilot trial | Intervention program |
| [35] McEvoy, Okekoya, Ivory, & Ivory (2016) | USA | School peers | School peers | Verbal and physical violence | 78 participants, 67.95% female | Mean = 19.74  Range/SD not stated. | Randomized control experiment | Intervention program |
| [38] Jouriles, Rosenfield, Yule, Sargent & Mcdonald (2016) | USA | Dating or potential dating relationship | Friends | Sexual assault | 80 students at a public high school in the southwestern US (42 female) | Range: 14~19 years  Mean = 15.8, SD= 1.20 | Not specified | Observational measure: simulated scenarios |
| [20] Jouriles, McDonald, Rosenfield & Sargent (2019) | USA | Dating or potential dating relationship | Friends | Sexual assault | 165 participants recruited from a low-income urban public high school in the southern US (51.5% female) | Range: 14~19  Mean = 15.7, SD =1.1 | Randomized control trial | Observational measure: simulated scenarios |
| [21] Jouriles, Kleinsasser, Rosenfield & McDonald (2016) | USA | Dating or potential dating relationship | Friends | Sexual assault | 91 undergraduate from social psychology classes at a midsize university in the southwestern US. (74 female) | Range: 18~23  Mean = 19.8, SD = 1.2 | Not specified | Observational measure: simulated scenarios |
| [36] Krauss et al., (2017) | USA | Dating or potential dating relationship | Friends | Sexual assault and relationship violence | 299 first-year students in required wellness classes at a midsized private university | Range: 18~21  Mean = 18.3, SD = 0.5 | Cross-sectional | Observational measure: simulated scenarios |
| [30] Sargent, Jouriles, Chmielewski & McDonald (2020) | USA | Friends, acquaintances, prior dating relationship | Friends | Sexual assault, physical violence, stalking, and coercive controlling behavior | 264(46% male) 1st-year US undergraduate students | Range  Mean=18.2, SD=0.6 | Randomized control trial | Observational measure: simulated scenarios |
| [29] Hortensius, Neyret, Slater & Gelder (2018) | Spain | Strangers (fans supporting different soccer teams) | Strangers (fans supporting the same soccer team) | Verbal and physical assault | 29 male supporters of F.C. Barcelona | Range: 18~29  Mean/SD not stated. | Experiment | Observational measure: simulated scenarios |
| [19] Rovira, Swapp, Spanlang & Slater (2009) | Not specified | Strangers (fans supporting different soccer teams) | Strangers (fans supporting the same soccer team) | Verbal and physical assault | 25 volunteers (no additional information) | Not reported. | pilot experiment | Observational measure: simulated scenarios |
| [37] Rovira, Swapp, Southern, Zhang & Slater (2013) | UK | Strangers (fans supporting different soccer teams) | Strangers (fans supporting the same soccer team) | Verbal and physical assault | 20 male supporters of English Premier League soccer team Arsenal F.C. | Range: 18~34  Mean/SD not stated. | Experiment | Observational measure: simulated scenarios |
| [31] Slater et al., (2013) | UK | Strangers (fans supporting different soccer teams) | Strangers (fans supporting the same soccer team) | Verbal and physical assault | 40 male supporters of Arsenal Football Club | Range: ≥18  Mean/SD not stated. | Two-factor between-group experiment | Observational measure: simulated scenarios |

**Table S2.** Summary of the research findings.

| Study | Research Question | Measurements | Summary of key findings | Strengths | Limitations |
| --- | --- | --- | --- | --- | --- |
| Ingram et al., (2019) | To examine whether VR enhanced intervention program correlates to decreased bullying behaviors, and whether empathy mediates the associations between the VR treatment and bullying behaviors, willingness to intervene, and school belonging? | (1) Empathy (Bosworth & Espelage, 1995); (2) School belonging (Goodenow, 1993); (3) Willingness to intervene; (4) Bully scale (Espelage & Holt, 2001); (5) Relational aggression perpetration (Crick, 1996);  (6) Cyberbullying perpetration (Ybarra et al., 2007) | (1) The treatment group reported significantly higher rates of empathy; (2) Empathy was associated with significant decreases in traditional bullying perpetration; (3) The mediation effect of empathy to reduce traditional bully was significant; (4) Empathy was associated with significant increases in willingness to intervene and school belonging; (5) The mediation effect of empathy on people’s willingness to intervene was significant. | (1) Validated the usefulness of VR as a novel approach in intervention design; (2) Participants found the VR experience realistic and meaningful. | (1) Small sample size; (2) Practical and ethical constraints limited a highly rigorous design; (3) No comparison between the VR-integrated and non-VR programs; (4) lack of cost analyses, practical challenges (charging, portability), sustained effect of the program. |
| McEvoy, Okekoya, Ivory, & Ivory (2016) | To examine whether interventions using VR more effective than interventions using less immersive electronic media such as videos | (1) Empathetic feelings (Batson et al. 1997); (2) Attitudes toward bullies and victims (Batson et al. 1997); (3) Attitudes toward bullying (Swearer et al., 2008); (4) future bystander intervention behavior in bullying situations (Banyard et al., 2007); (5) Yes-or-no if bullying is a problem in their school (Swearer et al., 2008). | (1) The quality of the VR graphics affected empathy; (2) The VR simulations evoked some feelings of presence and empathy; (3) The customization of the simulations was ineffective; (4) VR simulation was no more effective than video watching; (5) The bullying portrayed needed to match bullying that occurs within the target audience population. | Assessed the difference of effects between virtual reality simulations and effects of video and between customization of simulations | The two following findings contradict literature and were inconclusive: (1) VR is no more empathy evoking than video; (2) the customization of the VR experience to evoke similarity between the victim and bystander appears to be ineffective. |
| Jouriles, Rosenfield, Yule, Sargent & Mcdonald (2016) | To examine whether students’ feelings of responsibility, efficacy, and perceptions of benefits predict bystander behavior during simulated situations of dating violence | (1) Failure to take responsibility (Burn, 2008); (2) Bystander efficacy (Banyard et al., 2004); (3) Perceived benefits for intervening (Banyard et al., 2004); (4) decisional balance scale (Banyard et al., 2004) | Efficacy was positively associated with bystander behavior after controlling for responsibility and perceived benefits of intervening. | (1) VR as a novel method for assessing high-school students’ bystander behavior; (2) VR simulation circumvents some limitations of self-report questionnaires about bystander behavior (e.g., do not remember past situations accurately, use double-barreled questions that conflate bystander behavior with an opportunity to act as a bystander). | (1) The determinants not specifically designed for high-school students; (2) Limited number of situations; (3) Question of generalizability from VR to real-life; (4) Overlooked other possible determinants; (5) Small sample size |
| Jouriles, McDonald, Rosenfield & Sargent (2019) | To evaluate the effect of TakeCARE, a video bystander program, to prevent relationship and sexual violence among high school students. | (1) Bystander self-efficacy (Banyard et al., 2005); (2) Self-reported bystander behavior scale (Banyard et al., 2014); (3) Observed bystander behavior in VR simulations. | (1) The treatment group engaged in more bystander behavior in a 6-month follow-up; (2) Observed bystander behavior was greater in the TakeCARE condition at both the postintervention assessment and the 6-month follow-up. Females performed more bystander behaviors than males; (3) TakeCARE group reported greater self-efficacy than those in the control group. | (1) IVEs offer the opportunity to directly observe bystander behavior under experimentally controlled conditions; (2) The first evaluation of bystander programs that used both observational and self-report methods; (3) Longer follow-up period to measure the durability of TakeCARE’s effects. | (1) The obtained effect sizes remain unclear; (2) limited bystander scenario; (3) small sample size; generalizability. |
| Jouriles, Kleinsasser, Rosenfield & McDonald (2016) | To test the hypothesis that observed bystander behavior in the simulations would positively correlate with sense of responsibility, efficacy, behavioral intent and self-reported bystander behavior. | (1) Failure to take responsibility (Burn, 2008); (2) Bystander efficacy (Banyard et al., 2007); (3) Bystander attitudes (Banyard et al., 2007); (4) Bystander behavior scale (Banyard et al., 2005); (5) Simulation realism; (6) Adherence to the script. | (1) Bystander behavior in the simulations was positively correlated with bystanders’ responsibility for intervening, efficacy for intervening, intent to intervene, and self-reported bystander behavior at the 2-month follow up. (2) No effect of perceived realism of the simulations on observed bystander behavior | (1) New method evaluating bystander intervention programs, combining questionnaire and direct observations of behavior in IVE, which presented a more compelling measure for program effects, thus gain greater scientific credibility. | (1) IVE simulation was labor-intensive, expensive, time-consuming and not identical across administrators. Infeasible for large-scale evaluations compared to self-report questionnaires. (2) Limited simulation scenario. (3) Optimal duration is not clear (4) Small sample size. |
| Krauss et al., (2017) | To examine whether having adverse consequences of being an active bystander related to lower efficacy for intervening and less effective bystander behavior | (1) Self-reported negative consequences being a bystander (Banyard et al., 2004); (2) Bystander Efficacy Scale (Banyard et al., 2004); (3) Quality of bystander behavior (QBB) based on audio recording. | (1) Lower efficacy and QBB were associated with previous bystander behaviors (being physically hurt or getting into trouble as a result of trying to help); (2) Bystander training related to decreased negative consequences and higher QBB scores. | An initial attempt to examine the prevalence and correlates of previous negative consequences on later bystander behaviors. | (1) Cross sectional data; (2) Limited measures of adverse consequences; (3) Overlooked positive consequences of being an active bystander; (4) Small sample size. |
| Sargent, Jouriles, Chmielewski & McDonald (2020) | To examine whether VR simulations can be used to assess adolescent resistance to antisocial peer pressure | (1) Resistance to antisocial peer pressure in VR simulations; (2) Effectiveness of bystander inventions; (3) VR Immersion and Realism; (4) Peer Pressure (Santor et al., 2000); (5) Honest conduct (Hamby et al., 2013); (6) Dating violence (CADRI; Wolfe et al., 2001); (7) Depression (Radloff, 1977) | (1) VR resistance was associated with antisocial behavior and dating violence perpetration; (2) Female VR resistance scores were greater and dating violence perpetration was higher. | (1) allowed for standardized exposures across participants, which controlled for potential differences; confounds inherent in involving real-life peers were mitigated; (2) offered data that may not be accurately captured by self-reports; (3) offered a new tool to rigorously control and systematically manipulate the stimuli presented to measure Antisocial peer behavior. | (1) VR technology is not yet sufficiently scalable to be cost-effective for everyday clinical use; (2) limited peer pressure scenarios; (3) generalizability; (4) VR assessments cannot distinguish between the actively engaging in antisocial behavior from a lack of ability to resist peer pressure. |
| Hortensius, Neyret, Slater & Gelder (2018) | To examine whether reflexive and reflective behavioral responses to an emergency are related to later helping behavior in violent conflict | (1) Cued reaction time (Hortensius & Gelder, 2016); (2) Decision-making during the conflict (Rand & Epstein, 2014); (3) Interpersonal reactivity index (Davis, 1980; Davis, 1983; Perez-Albeniz et al., 2003); (4) Presence (Sanchez-Vives & Slater, 2005; Slater & Steed, 2000; Pan et al., 2015). | (1) Faster responses to an emergency in low cognitive load condition was significantly associated with increased helping behavior during the violent conflict simulation. (2) Participants tend to report their decision to intervene as intuitive and reflexive provided more help. (3) Sympathy was related to self-reported intuitive decision-making style, a decreased distance to the aggressor, higher in the intervening participants. | VR made it especially possible to measure phenomenological, behavioral and physiological reactions during situations that were part of everyday life. | (1) Generalizability; (2) No gender differences tested; (3) Measures of helping behavior in a virtual world; (4) Small sample size. |
| Rovira, Swapp, Spanlang & Slater (2009) | To examine the use of VR to study bystander responses to violent incidents | (1) Number of participants intervening in the conflict  (2) Qualitative interview: (a) General experience (b) Did you look around to look for other people? (c) What would have made it more likely for you to intervene? | (1) 7/13 in the glance condition and 4/12 in the non-glance group intervened. (2) 10/25 looked around to see if someone else was in the scenario  (3) People are less likely to intervene if they know (from a technical point of view) that their intervention cannot achieve anything. (4) People become involved in the scenario, and many who did not intervene in thoughts about intervention or their safety. | (1) Better generalizability than lab-based actions or opinion-based measurement (2) People tend to have a range of realistic responses in VR | Ethical concerns: (1) Cause stress to participants; (2) "Desentisation" about aggressive acts.  Tech concerns: (1) The credibility of the scenario; (2) "Many aspects of the simulation can be technically wrong" |
| Rovira, Swapp, Southern, Zhang & Slater (2013) | To examine the impact of display resolution and luminance of the IVR system on participants’ responses | Number of physical (e.g., moving close to avatars) and verbal interventions. Feelings and thoughts during the confrontation. | Significant higher numbers of both physical and verbal interventions in the post-upgrade version of the scenario compared to the pre-upgrade group. | Impact of technology and different set-ups on participants’ responses. | Not stated. |
| Slater et al., (2013) | To test the hypothesis that the psychological relationships between bystanders and others (e.g., victims) – group identification is important for bystander behaviors | (1) Numbers of Interventions; (2) Post-questionnaire, including uncomfortable, othersafety, ownsafety, otherpeople and victimlooked; (3) interview questions, such as wanted to stop it, anger, anxiety to fear, confrontational. | (1) The number of physical interventions was higher in the in-group than those in the out-group. (2) In the in-group, the more that participants perceived that the victim was looking towards participants for help the greater the number of physical interventions. (3) Physical interventions related to the safety of the victims; verbal interventions were more likely to relate to their safety. | (1) Findings are generalizable; (2) Genetic programming exploring the data deeply than normal statistical models. | (1) The interactivity and credibility of the scenario can be improved; (2) Validity of people’s responses to the virtual situation. |

**Table S3.** Summary of virtual reality/augmented reality designs.

| Study | VR/AR design/name/company | Time scheduled for VR/AR | Description of the Scenario |
| --- | --- | --- | --- |
| Ingram et al., (2019) | (1) Custom VR scenarios (Polanin et al., 2012); (2) Daydream Goggle (Daydream. (n.d.). | (1) The virtual reality scenarios took approximately 5 minutes per week for 3 weeks; (2) Total time in VR scenario: 15mins/ participant | Three VR scenarios were used: (1) where a student was bullied and became an outcast at school. Then, when his only friend was the victim of bullying and relational aggression, he participated. The victim sought help and the friend stood up for her amongst his new popular friends; (2) three short scenes that showed adults delivering different ineffective responses to bullying; (3) participants time-travel to a future where bullying no longer exists. Peers from the future explain how bullying became extinct and teach the time-travelers how to intervene. |
| McEvoy, Okekoya, Ivory, & Ivory (2016) | (1) Simulation created from the “Be More than a Bystander” campaign video in Unity; (2) Delivered by Oculus Rift. | (1) Video condition is 30 seconds long; (2) VR scenario uses the same audio and assumed to be also 30 seconds long. | Participants in the VR groups were immersed in VR experiences based on the same video as the video group, the customized group experienced the victims wearing the same identifying clothing as themselves. The VR scenarios feature the participant seeing a female student being verbally and physically bullied by two other female students in the school hallway near the lockers between classes. |
| Jouriles, Rosenfield, Yule, Sargent & Mcdonald (2016) | Viewed through goggles (brand not stated) | (1) Two rounds of VR simulations: at 1-week and 6-month after the baseline assessment; (2) 9 simulations for each round: 4 experimental and 5 distractors; (3) Each simulation was 2 to 4 min. | In each simulation, the IVE would display the inside of a parked car and participants are seated as a passenger, together with the driver - a male student friend (avatar), controlled by a trained male actor via computer. The scene began with a research assistant providing background information. The actors would then go through nine scripted statements, one to two of which suggested imminent potential for violence. Specific simulation scenarios were the same as the research below. |
| Jouriles, McDonald, Rosenfield & Sargent (2019) | Viewed through goggles (brand not stated) | (1) Two rounds of VR simulations: postintervention and 6-month follow-up assessment; (2) 9 simulations for each round: 4 experimental and 5 distractors; (3) Each simulation was 2 to 4 min. | In each simulation, the IVE would display the inside of a parked car and participants are seated as a passenger, together with the driver - a male student friend (avatar), controlled by a trained male actor via computer. The scene began with a research assistant providing background information. The actors would then go through nine scripted statements, one to two of which suggested imminent potential for violence. Four specific simulation scenarios are:(1)Drunk night – participant being informed about an unconscious friend being taken into a back bedroom at a party; (2)Stormy relationship – the avatar pointing out to the participant a situation in which a friend has just thrown her book at her boyfriend in a heated argument; (3)Homecoming dance – the avatar talking about conducting a potential unwanted sexual activity toward the date who refused his previous sexual advances; (4)The hook-up - the avatar describing plans to return to a party to hook up with a drunk woman. |
| Jouriles, Kleinsasser, Rosenfield & McDonald (2016) | Viewed through goggles (brand not stated) | (1) 7 simulations for each round: 3 experimental and 4 distractors  (2) Each simulation was 2 to 4 min | In each simulation, the IVE would display the inside of a parked car and participants are seated as a passenger, together with the driver - a 25-year-old male student friend (avatar), controlled by a trained male actor via computer. The scene began with a research assistant providing background information. The actors would then go through 7 to 8 scripted statements. (1) Intoxicated woman at party - participant being informed about an unconscious friend being taken into a back bedroom at a party. (2) Going back to hook up - the avatar describing plans to return to a party to hook up with a drunk woman. (3) Fraternity formal - the avatar talking about conducting a potential unwanted sexual activity toward the date who refused his previous sexual advances. |
| Krauss et al., (2017) | Viewed through Oculus Rift goggles | (1) Total of 5 simulations: 3 experimental simulations and 2 distractor simulations (2) Each simulation was 2 to 4 min | In each simulation, participants are seated in a parked car as a passenger, together with the driver - a male student friend (avatar), controlled by a trained male actor via computer. The scene began with a research assistant providing background information. The actors would then go through nine scripted statements, one to two of which suggested imminent potential for violence. The 3 Bystander simulations included the avatar telling the participant about an unconscious friend being taken into a back bedroom at a party (B-Sim1), the avatar pointing out to the participant a situation in which a friend has just thrown her backpack at her boyfriend in a heated argument (B-Sim2), and the avatar describing plans to return to a party to hook up with a drunk woman (B-Sim3). |
| Sargent, Jouriles, Chmielewski & McDonald (2020) | (1) Custom VR application. (2) Delivered through Oculus Rift goggles (by author) | (1) 2 mins VR simulation per assessment; (2) Total time of 18mins VR simulation for each participant; (3) half the sample repeated the VR simulations at a 2 months follow-up. | Participants experienced themselves as seated in the passenger seat of a parked car, with a peer (avatar) in the driver’s seat. Actors engaged participants in a conversation with five to six scripted statements. Four simulations included pressure from the avatar to engage in antisocial or risky behavior, five involved situations of actual or potential relationship aggression. At least two scripted statements in each peer-pressure simulation directly pressured the participant to engage in or facilitate antisocial behavior. |
| Hortensius, Neyret, Slater & Gelder (2018) | (1) XVR programming platform (Tecchia, 2010); (2) the virtual characters animated with HALCA software (Gillies & Spanlang, 2010); (3) delivered by a CAVE system (Cruz-Neira et al.,1992); (4) head tracker (InterSense IS900); (5) Crystal Eyes shutter glasses | (1) Normal conversation between the participant and the victim: 103 ± 24s; (2) Total time of conflict between the victim and the perpetrator: 135s | The participants had a short free-flow conversation with the virtual human (victim) whose utterances had been prerecorded to ensure it's a normal conversation. The virtual human (perpetrator) started an argument with the virtual victim and then escalated into a physical attack. V took a submissive role and made eye contact with the participant. The conflict between P and V was the same for all participants. |
| Rovira, Swapp, Spanlang & Slater (2009) | Delivered by a Trimension Reactor system | A brief introduction between the participant and the virtual victim. 2 min and 20 seconds of conflict. | Perpetrator becomes increasingly threatening until finally the perpetrator begins to push Victim against the wall. In terms of body size, gestures and also voice tone, and that V is submissive and wanting to avoid trouble. However, whatever answer is given by V, P uses this to escalate the argument to a more dangerous level. Both characters are life-sized. |
| Rovira, Swapp, Southern, Zhang & Slater (2013) | (1) Cave-like VR system (Cruz-Neira et al.,1993). (2) Pre-upgrade setup: 4 CRT projectors 1024×768 @90Hz and 1250 Lumens. (3) Post-upgrade setup: 4 DLP projectors 1400×1050 @100Hz and 3150 Lumens. | The confrontation was 2’ 12”. The total length of VR simulation is ~7 minutes, depending on the length of the conversation with the virtual man before the confrontation. | Participant is approached by a virtual man (Victim) wearing an Arsenal red shirt who attempts to bond over support of Arsenal. A second virtual man (perpetrator) starts to verbally attack Arsenal. The argument escalates. The experiment ends when it gets to the point of physical violence. The confrontation is scripted and always the same. |
| Slater et al., (2013) | (1) Delivered by a CAVE system (Cruz-Neira et al.,1992); (2) Crystal Eyes shutter glasses; (3) InterSense IS-900 head-tracking device; (4) XVR programming platform (Tecchia et al., 2010); (5) Hardware accelerated library for character animation (Gillies & Spanlang, 2010) | Total 6.3 mins (2 mins look around for items related to football; 2 mins talk to the victim; 140 s perpetrator interact with the victim) | The participant is left alone in the bar having been instructed to look around for items related to football for 2 minutes. A virtual character (victim) enters the scene and starts a conversation. A virtual human (perpetrator) starts an argument with the virtual victim and then escalates into a verbal and physical attack. In the LookAt condition, the virtual human (victim) looks toward the participants 5 times for 3 seconds each. |
